# H. Pylori is related to osteoporosis but only in premenopausal female: a cross-sectional study

**DOI:** 10.1186/s12891-020-03586-7

**Published:** 2020-08-18

**Authors:** Jing-Wei Wang, Feng-Xiao Dong, Hui Su, Licun Zhu, Sujun Shao, Hong Liu

**Affiliations:** 1 Department of Gastroenterology, Beijing Shijitan Hospital, Capital Medical University, No. 10, Tieyi Road, Beijing, China; 2 Department of Physical examination center, Beijing Shijitan Hospital, Capital Medical University, No. 10, Tieyi Road, Beijing, China; 3 Department of Gastroenterology, Beijing Shijitan Hospital, Capital Medical University Beijing, Beijing, 100038 China

**Keywords:** Female, H. Pylori infection, Osteoporosis, Premenopausal, Chronic inflammation

## Abstract

**Background:**

Recently, an increasing number of studies have focused on the extra-gastrointestinal effects of Helicobacter pylori (H. pylori), including metabolic syndrome, fatty liver, and rheumatic and skin diseases. Osteoporosis is an asymptomatic disease that can eventually lead to fractures and has a significant impact on the quality of life of elderly individuals. Sex is an influential factor that plays a crucial role in the development of osteoporosis. The aim of this study was to investigate the relationship between H. pylori infection and osteoporosis and to identify potential influencing factors.

**Methods:**

We conducted a cross-sectional study of individuals older than 50 years old, who had undergone regular physical examinations at the Beijing Shijitan Hospital Health Examination Center from July to October 2018. We evaluated the associations of osteopenia and osteoporosis with H. pylori infection and related serum markers by using multiple linear regression and logistic regression. Then, we analysed the correlation between sex and potential serum biomarkers.

**Results:**

There were significant relationships between H. pylori infection status and bone density in premenopausal females but not in males (*P* = 0.381) according to Fisher’s exact test. In females, H. pylori positivity (OR = 0.132, *P* = 0.023), Body Mass Index (BMI) (OR = 28.163, *P* = 0.021), and homocysteine (HCY) (OR = 17.218, *P* = 0.045) were associated with osteoporosis. Calcium had a trend but no statistically significant (OR = 0.060, *P* = 0.076) relationship with osteoporosis. Furthermore, the waist-to-hip ratio (OR = 5.783, *P* = 0.029), BMI (OR = 0.152, *P* = 0.014) and triglyceride levels (OR = 0.201, *P* = 0.036) were significantly different by sex, after adjusting for age as a confounder.

**Conclusion:**

H. pylori positivity, BMI and HCY are associated with osteoporosis in premenopausal females. Chronic inflammation may be involved in the relationship between H. pylori and osteoporosis.

## Background

Helicobacter pylori (H. pylori), a gram-negative, spiral-shaped microaerophilic bacterium, has been shown to be an important pathogen in gastrointestinal diseases [1]. Approximately 50% of the world population has been affected by H. pylori, and approximately 800 million Chinese individuals are affected by this disease. It may cause chronic inflammation of the gastric mucosa, which may lead to chronic atrophic gastritis, peptic ulcer diseases, and gastric cancer [2, 3]. Moreover, the latest reports have described the investigation of the extra-gastrointestinal effects of H. pylori, including metabolic syndrome [4], fatty liver [5], and rheumatic and skin diseases [6]. These parenteral diseases associated with H. pylori infection seriously affect the patient’s general condition and cause a series of complications.

Osteoporosis is an asymptomatic disease characterized by a decreased density of normally mineralized bone that usually occurs in elderly persons. It has been reported that approximately 2 million men and 8 million women over the age of 50 in the United States have been diagnosed with osteoporosis, with an estimated 34 million suffering from osteopenia [7]. In the development of osteoporosis, there is often a long latent period before the appearance of the main clinical manifestation: pathologic fractures. Moreover, the most prevalent sequelae of osteoporosis are compression fractures of the vertebral bodies and fractures of the ribs, proximal femurs, humeri, and distal radiuses, which could have a significant impact on the quality of life of elderly individuals. Therefore, the prevention and early detection of osteoporosis is particularly important for the elderly population.

A majority of studies support the idea that at any given age, women have a higher risk of fracture than men [8]. However, men tend to have worse outcomes after fracture than women: they are twice as likely to die after a hip fracture than women [9]. Moreover, sex-related factors remain unclear. Therefore, exploring the differences in factors influencing osteoporosis in males and females will be helpful to explore the pathogenesis of osteoporosis and early prevention of its occurrence and development.

There are some well-established risk factors for the emergence of osteoporosis, such as age, sex, body mass index, and alcohol consumption [10]. Recent articles have focused on H. pylori and osteoporosis. Different researchers have proposed different theories, including but not limited to, inflammation induced by H. pylori infection [11] and malabsorption of nutrients [12, 13]. However, there is still a lack of systematic data analyses on the relationship between H. pylori and osteoporosis. The association between osteoporosis and H. pylori has been studied by many Japanese scientists and remains controversial. In addition, there are still some deficiencies in existing research. Few studies have focused on the correlation between H. pylori and osteoporosis in Chinese premenopausal females. The sample sizes have been insufficient, there is a lack of investigations on the influence of sex, and there is a lack of sufficient serum markers. The aim of this study was to investigate the relationship between H. pylori and osteoporosis and to identify potential influencing factors.

## Methods

### Study population

Briefly, men and women, older than 50 years old (i.e., not including those aged 50 years old), who had regular physical examinations at the Beijing Shijitan Hospital Health Examination Center from July to October 2018 were included. Our research used the following exclusion criteria for the data collection periods: (1) patients using the following drugs and having comorbidities that may cause secondary osteosis: glucocorticoids, thyroid/parathyroid drugs, psychotropic drugs, anticonvulsants, selective estrogen receptor modulators (SERMs), vitamin D, calcium, and bisphosphonate; (2) patients who had a history of gastrectomy, inflammatory bowel disease, malignant diseases, chronic kidney disease, diabetes mellitus, hypo/hyperthyroidism, hypo/hyperparathyroid disorder, acromegaly and rheumatoid arthritis (including collagen disease). Study participants who had been diagnosed with H. pylori infection before or had potentially anti-H. pylori drugs in the past 1 month were recruited as well.

We also excluded postmenopausal women in the present study. The criteria for determining menopause, based on the latest guidelines, included any of the following: (1) prior bilateral oophorectomy; (2) age ≥ 60 years old; (3) age < 60 years and amenorrhoeic for 12 or more months in the absence of chemotherapy, tamoxifen, or toremifene [14]. The female study participants were not pregnant or lactating.

### Data collection

Among all the eligible individuals, 228 eligible study participants gave informed verbal consent and provided their basic information, including demographics (age, sex, race), smoking status, and medicine use. All ethics approvals were given by the Ethics Committee of Beijing Shijitan Hospital affiliated with Capital Medical University, and the study was performed in accordance with the Declaration of Helsinki. Blood measurements were performed with fresh serum obtained after a 12-h fast to minimize the confounding effects of diurnal variation on hormone concentrations and included tests for glucose metabolism, liver function, renal function, lipid metabolism, ions (calcium, iron), tumour markers, pepsinogen (PG), and progastrin-releasing peptide (proGRP). Anthropometric measurements, including waist circumference (cm), blood pressure (mmHg), body weight (kg) and height (cm), were measured by trained nurses using a standardized protocol. Diastolic and systolic blood pressure were measured in the morning. Body mass index (BMI) was calculated by taking a person’s weight in kilograms divided by their height in metres squared. The waist-to-hip ratio (WHR) is measured as waist circumference divided by hip circumference. H. pylori infection status was measured by a [13]C breathing test on the same day with an empty stomach. Tumour markers were measured using enzyme-linked immunosorbent assay methods.

### Diagnosis of osteoporosis

The bone mineral density (BMD) of lumbar vertebrae 2–4 (L2–4) was measured by DXA using a Discovery DXA system (Hologic, Bedford, Massachusetts). The results provided BMD (g/cm2) and young adult mean bone mineral density. The diagnosis of osteoporosis was performed in accordance with the World Health Organization diagnostic criteria from the World Health Organization (WHO) Collaborating Center for Metabolic Bone Diseases [15]. A value for BMD within one standard deviation (SD) of the average BMD of normal adults was regarded as normal. Osteopenia is defined as a BMD that lies between 1 and 2.5 standard deviations below the young adult mean value. BMD more than 2.5 SD below the young adult mean value was classified as osteoporosis [15].

### Statistical analyses

We used SPSS statistical software version 22.0 for data analyses. Continuous variables were reported as means ± standard deviations, whereas categorical variables were presented as percentages. Study subjects were first classified into three groups according to BMD classification: normal, osteopenia, and osteoporosis. The Kolmogorov-Smirnov test was used to verify whether the data fit a normal distribution, and all continuous variables that did not conform to a normal distribution underwent transformation for analysis. Summary and grouping data for baseline characteristics (the laboratory examination) were compared using a t test for continuous variables and Fisher’s exact test for categorical variables in the H. pylori- and H. pylori+ groups. Moreover, we divided the study population by sex and used Fisher’s exact test to verify whether there were sex differences in the relationship between H. pylori and osteoporosis.

Multivariate-adjusted odds ratios (ORs) and 95% confidence intervals (CIs) were calculated using logistic regression among the three subgroups. To further analyse the relationship between H. pylori infection status and osteoporosis, we created a model using total cholesterol (TC), triglycerides (TG), uric acid (UA), BMI, WHR, low-density lipoprotein cholesterol (LDL-C), C-reactive protein (CRP), homocysteine (HCY), H. pylori infection status, calcium (Ca), vitamin B12 and the BMD groups that analysed the different sexes separately. The study population’s high-density lipoprotein cholesterol (HDL-C) and glucose levels were all normal, so we did not include them in the model.

Meanwhile, we analysed the differences in markers between males and females. We separated the study population according to sex and further analysed the patients’ basic data in the same way as we analysed the baseline characteristics. We further analysed the relationship between sex and markers to find sex differences in the relationship between H. pylori infection and osteoporosis. The markers included TC, LDL-C, UA, TG, glucose (GLU), CA724, CEA, BMI, SP (systolic blood pressure), BP (diastolic blood pressure), WHR, HCY, CRP, vitamin B12, Ca, and Ghb. Other serum biomarkers were entered into the model as factors using their normal value as the grouping criterion. All the models were adjusted for age as a confounder. A two-tailed *P*-value < 0.05 was considered to be statistically significant.

## Result

The baseline characteristics of the participants are shown in Table 1. The mean age was 54.53 ± 0.238 years, 167 (73.2%) were male, and 76 (26.8%) were female. Among the study population, 40 (17.5%) had osteoporosis, 77 (33.8%) had osteopenia, and 111 (48.7%) had normal BMDs. The mean ages of the osteoporosis, osteopenia and normal groups were 53.43 ± 2.836, 54.53 ± 3.589 and 53.98 ± 3.205, respectively. There were no significant differences in age among the participants in the osteoporosis, osteopenia and normal BMD groups. Fifty percent of the males had normal BMDs, 34.7% of the males had osteopenia, and 15.0% of the males had osteoporosis. In addition, 44.3% of the females had normal BMDs, 31.1% of the females had osteopenia, and 24.6% of the females had osteoporosis. There was no significant difference in sex distribution among these three groups.

**Table 1:** Baseline Characteristics of the patients according to the H. pylori infection statue

		Total	H. pylori-	H. pylori+	*P-value*
Age		54.13 ± 3.30	54.02 ± 3.63	54.02 ± 3.63	0.887
Sex	Female	26.8%	65.6%	34.4%	0.834
	Male	73.2%	64.1%	35.9%	
BMD	normal	48.7%	48.3%	49.4%	0.912
	osteopenia	33.8%	34.7%	32.1%	
	osteoporosis	17.5%	17.0%	18.5%	
SP (mmHg)		123.20 ± 16.802	125.96 ± 17.772	125.96 ± 17.772	0.065
BP (mmHg)		75.53 ± 12.494	76.74 ± 12.350	76.74 ± 12.350	0.313
BMI (kg/m^2^)		24.752 ± 3.1597	24.964 ± 3.350	24.964 ± 3.350	0.418
WHR		0.901 ± 0.051	0.902 ± 0.049	0.902 ± 0.050	0.920
Hb(g/L)		149.51 ± 13.397	149.41 ± 13.825	149.41 ± 13.825	0.798
Platelet(*10^9^/L)		236.97 ± 51.960	234.05 ± 46.746	234.05 ± 46.746	0.599
Ca (mmol/L)		2.313 ± 0.070	2.313 ± 0.070	2.313 ± 0.075	0.973
UA (umol/L)		365.61 ± 81.545	365.37 ± 85.073	366.05 ± 75.083	0.910
TC (mmol/L)		4.996 ± 0.875	4.967 ± 0.841	5.051 ± 0.938	0.461
TG (mmol/L)		1.611 ± 0.920	1.605 ± 0.947	1.621 ± 0.874	0.859
HDL-C (mmol/L)		1.292 ± 0.286	1.293 ± 0.284	1.291 ± 0.292	0.911
LDL-C (mmol/L)		2.685 ± 0.641	2.661 ± 0.615	2.731 ± 0.688	0.354
GLU (mmol/L)		5.519 ± 1.390	5.392 ± 1.213	5.754 ± 1.653	0.063
CEA (ng/ml)		2.549 ± 1.557	2.430 ± 1.438	2.771 ± 1.744	0.103
CA724(U/ml)		3.543 ± 4.854	4.103 ± 5.725	2.536 ± 2.388	**0.025**
Ghb(%)		5.838 ± 1.103	5.767 ± 0.725	5.966 ± 1.545	0.307
Iron (umol/L)		19.23 ± 5.667	19.210 ± 0.578	19.27 ± 5.881	0.952
CRP (mg/L)		1.82 ± 2.430	1.90 ± 2.557	1.69 ± 2.199	0.626
HCY (umol/L)		16.187 ± 7.497	16.518 ± 8.605	15.592 ± 4.937	0.486
B12(pmol/L))		357.61 ± 185.280	360.07 ± 190.901	353.20 ± 176.515	0.834

*WHR* Waist-to-hip ratio; *TC* Total cholesterol; *Ghb* Glycosylated hemoglobin; *TG* Triglyceride; *UA* Uric acid; *AST* Aspartate aminotransferase; *ALT* Alanine aminotransferase; *Ca* Calcium; *Cre* Creatinine; *DP* Diastolic blood pressure; *Hb* Hemoglobin; *HDL-C* High-density lipoprotein cholesterol; *LDL-C* Low-density lipoprotein cholesterol; *BMD* Bone mineral density; *OR* Odds ratio; *proGRP* Pro-gastrin-releasing peptide; *WHR* Waist-to-Hip Ratio; *SP* Systolic blood pressure; *PG* Pepsinogen; *GLU* Glucose; *CRP* C-reactive protein; *HCY* HOMOCYSTEINE;

Bold indicates statistically significant values

Among all the study participants, 35.5% had H. pylori infections, and 64.5% did not have H. pylori infections. Table 1 shows that CA724 (t = 2.265, *P* = 0.025) was significantly different between the study participants with H. pylori infection and those without H. pylori infection, and there were no significant differences between the H. pylori infection status and the BMD status groups.

In Table 2, we separate the study population according to sex. We found that there was a significant relationship between H. pylori infection status and bone density in females (*P* = 0.025) but not in males (*P* = 0.381).

**Table 2:** the relationship between the H. pylori infection and the BMD in different gender

Female			
	H. pylori infection (−)	H. pylori infection (+)	*P-value*
Normal BMD	18	9	**0.025**
osteopenia	16	4	
osteoporosis	6	9	
Male			
	H. pylori infection (+)	H. pylori infection (−)	*P-value*
Normal BMD	53	31	0.381
osteopenia	35	23	
osteoporosis	19	6	

*BMD* Bone mineral density;

Bold indicates statistically significant values

As shown in Table 3, there was a significant association of H. pylori positivity (OR = 0.132, 95% CI 0.023–0.753, *P* = 0.023), BMI (OR = 28.163, 95% CI 1.647–481.545, *P* = 0.021), and HCY (OR = 17.218, 95% CI 1.061–279.779, *P* = 0.045) with osteoporosis in premenopausal females. Ca (OR = 0.060, 95% CI 0.003–1.347, *P* = 0.076) and TC (OR = 7.250, 95% CI 0.773–68.033, *P* = 0.076) had a trend, but neither was significantly associated with osteoporosis.

**Table 3:** Multivariable analysis for different markers and BMD

	OR	95%CI	*P-value*
TC (mmol/L)			
Q1(3.65–5.20)	7.250	0.773,68.033	0.083
Q2(5.21–7.66)	0	0	
BMI (kg/m^2^)			
Q1(17.9–23.9)	28.163	1.647,481.545	**0.021**
Q2(24.0–31.9)	0	0	
UA (umol/L)			
Q1(187–357)	0.324	0.028,3.827	0.371
Q2(358–571)	0	0	
TG (mmol/L)			
Q1(0–1.70)	0.162	0.009,2.858	0.214
Q2(1.71–4.47)	0	0	
LDL-C (mmol/L)			
Q1(0–3.12)	0.273	0.029,2.524	0.252
Q2(3.13–4.20)	0	0	
C13			
Q1(without H. pylor)	0.132	0.023,0.753	**0.023**
Q2(with H. pylor)	0	0	
WHR			
Q1(0.79–0.84)	3.529	0.472,26.390	0.219
Q2(0.85–0.97)	0	0	
HCY			
Q1(0–14.9 μmol/L)	17.218	1.061,279.779	**0.045**
Q2(> 15 μmol/L)	0	0	0
CRP			
Q1 (0–4.9 mg/L)	0.167	0.001,32.917	0.507
Q2 (> 5 mg/L)	0	0	
Ca			
Q1 (2.1–2.25 mg/L)	0.060	0.003,1.347	0.076
Q1 (> 2.25 mg/L)	0	0	
B12			
Q1(0-516pmol/L)	0.228	0.706,54.927	0.100
Q2(> 516 pmol/L)	0	0	

Abbreviations as in Table 1; *OR* Odds ratio; *C13* ^13^C breathing test positive;

Bold indicates statistically significant values

Meanwhile, we analysed the differences in markers between males and females, which are shown in Table 4. We found that age (*P* < 0.001), SP (*P* < 0.001), BP (*P* < 0.001), BMI (*P* < 0.001), WHR (*P* < 0.001), Hb (*P* < 0.001), platelets (*P* < 0.001), UA (*P* < 0.001), TC (*P* = 0.002), TG (*P* = 0.006), HDL-C (*P* < 0.001), GLU (*P* = 0.002), CEA (*P* < 0.001), Ghb (*P* = 0.029), iron (*P* < 0.001), HCY (*P* < 0.001), and B12 (*P* = 0.002) demonstrated significant differences between males and females.

**Table 4:** Baseline Characteristics of the patients in different gender

	Total	Female	Male	*P-value*
Age	54.13 ± 3.304	52.23 ± 1.865	54.74 ± 3.441	**0.000**
SP (mmHg)	123.20 ± 16.802	112.79 ± 15.598	126.87 ± 15.767	**0.000**
BP (mmHg)	75.53 ± 12.494	66.05 ± 11.273	79.06 ± 10.966	**0.000**
BMI (kg/m^2^)	24.752 ± 3.1597	23.016 ± 3.104	25.336 ± 2.975	**0.000**
WHR	0.901 ± 0.051	0.880 ± 0.039	0.911 ± 0.053	**0.001**
Hb(g/L)	149.51 ± 13.397	135.79 ± 10.965	154.80 ± 9.995	**0.000**
Platelet(*10^9^/L)	236.97 ± 51.960	255.21 ± 51.091	229.67 ± 47.700	**0.001**
Ca (mmol/L)	2.313 ± 0.070	2.317 ± 0.081	2.311 ± 0.064	0.622
UA (umol/L)	365.61 ± 81.545	307.05 ± 67.596	386.49 ± 73.515	**0.000**
TC (mmol/L)	4.996 ± 0.875	5.335 ± 0.899	4.868 ± 0.835	**0.000**
TG (mmol/L)	1.611 ± 0.920	1.331 ± 0.754	1.706 ± 0.963	**0.006**
HDL-C (mmol/L)	1.292 ± 0.286	1.453 ± 0.267	1.235 ± 0.274	**0.000**
LDL-C (mmol/L)	2.685 ± 0.641	2.794 ± 0.638	2.636 ± 0.631	0.096
GLU (mmol/L)	5.519 ± 1.390	5.153 ± 0.742	5.631 ± 1.519	**0.002**
CEA (ng/ml)	2.549 ± 1.557	1.934 ± 1.119	2.768 ± 1.632	**0.000**
CA724(U/ml)	3.543 ± 4.854	3.742 ± 3.209	3.449 ± 5.477	0.740
Ghb(%)	5.838 ± 1.103	5.618 ± 0.424	5.942 ± 1.296	**0.029**
Iron (umol/L)	19.23 ± 5.667	16.90 ± 5.521	20.33 ± 5.421	**0.001**
CRP (mg/L)	1.82 ± 2.430	1.52 ± 1.626	1.97 ± 2.732	0.302
HCY (umol/L)	16.187 ± 7.497	12.887 ± 2.947	17.751 ± 8.448	**0.000**
B12(pmol/L))	357.61 ± 185.280	439.11 ± 221.573	319.01 ± 151.975	**0.002**

Abbreviations as in Table 1; *OR* Odds ratio;

Bold indicates statistically significant values

The relationship between sex and markers is shown in Table 5. We found that WHR (OR = 5.783, 95% CI 1.192–28.078, *P* = 0.029), TG (OR = 0.201, 95% CI 0.045–0.902, *P* = 0.036), and BMI (OR = 0.152, 95% CI 0.034–0.688, *P* = 0.014) were significantly different by sex, adjusting for age as a confounder.

**Table 5:** Multivariable analysis for different markers and gender

	OR	95%CI	*P-value*
WHR			
Q1(0.79–0.84)	5.783	1.192,28.078	**0.029**
Q2(0.85–0.97)	0	0	
TC (mmol/L)			
Q1(0–5.20)	1.694	0.345, 8.298	0.516
Q2(> 5.21)	0	0	
LDL-C (mmol/L)			
Q1(0–3.12)	2.502	0.374, 16.710	0.344
Q2(3.13–4.52)	0	0	
UA (umol/L)			
Q1(187–357)	1.096	0.246, 4.884	0.904
Q2(358–571)	0	0	
TG (mmol/L)			
Q1(0–1.70)	0.201	0.045, 0.902	**0.036**
Q2(1.71–13.19)	0	0	
GLU (mmol/L)			
Q1(0–6.10)	0.584	0.072,4.740	0.615
Q2(6.11–13.03)	0	0	
CA724(U/ml)			
Q1(0–6.89)	2.787	0.481, 16.151	0.253
Q2(6.90–39.18)	0	0	
CEA (ng/ml)			
Q1(0–4.99)	0.211	0.013, 3.511	0.278
Q2(5.00–9.85)	0	0	
BMI (kg/m^2^)			
Q1(17.9–23.9)	0.152	0.034,0.688	**0.014**
Q2(24.0–34.1)	0	0	
SP (mmHg)			
Q1(90–140)	0.133	0.005,3.228	0.215
Q2(> 140)	0	0	
BP (mmHg)			
Q1(60–90)	1.119	0.057,21.889	0.914
Q2(> 90)	0	0	
HCY (μmol/L)			
Q1(0–14.9)	0.377	0.091,1.559	0.178
Q2(> 15)	0	0	
CRP (mg/L)			
Q1 (0–4.9)	4.076	0.289,57.397	0.298
Q2 (> 5)	0	0	
B12(pmol/L)			
Q1(0–516)	6.038	0.681,53.464	0.106
Q2(> 516)	0	0	
Ca (mg/L)			
Q1 (2.1–2.25)	1.502	0.327,6.903	0.601
Q1 (> 2.25)	0	0	
Ghb(%)			
Q1(0–6.0)	0.487	0.064,3.691	0.487
Q2(6.1–7.3)			

Abbreviations as in Table 1; *OR* Odds ratio;

Bold indicates statistically significant values

Male and female have different normal value in UA and WHR, UA 149-416umol/L in male,89-357umol/L in female, WHR < 9.0 in male,< 8.5 in female

## Discussion

Osteoporosis is an important health and societal burden in elderly people, not only in females but also in males. There are numerous osteoporosis-related fracture risk factors, including age, sex, race, lifestyle and concomitant medical conditions [16]. In men, osteoporosis is underrecognized and undertreated. Only a few men are screened for osteoporosis, even after a fracture [17]. The treatment rate is much lower in males than in females [18]. Meanwhile, more men than women die every year due to hip fractures [19]. Hence, we also included men in the study population to determine the risk factors for osteoporosis.

Some studies about the influence of sex on osteoporosis remain controversial. In our study, there was a significant relationship between H. pylori and osteoporosis in premenopausal females but not in males. The reasons for the difference between males and females are as follows: First, differences in clinical outcomes of osteoporosis in men and women may be rooted in the biologic properties of bone. Barrett-Connor E holds the view that there are sex-specific differences in the number of osteoprogenitor cells and in hormone responses and regulation [20, 21]. Second, men have a greater bone size, trabecular BMD and bone area at the radius and tibia than women, even after adjusting for weight and height, which may lead to a decrease in osteoporosis and fracture [22]. Third, men undergo a slow decrease in BMD with increasing age, while women experience a profound period of rapid bone resorption, especially after entering menopause [23]. Last but not least, some studies support the idea that men are more likely to suffer from secondary disease, for example, rheumatoid arthritis, alcoholism, excessive smoking, gonadal deficiencies and others [24], which may lead to sustainable bone loss.

Unfortunately, the relationship between osteoporosis and H. pylori infection is still controversial. Some studies hold the view that there is no difference between men and women in the relationship between H. pylori and osteoporosis [25, 26]. Some studies hold the view that H. pylori is related to osteoporosis only in women. Shih-Chun Lin conducted a retrospective study including 365 women and showed that H. pylori is related to osteoporosis in females [27], while others think that there is no correlation between them in females. Daisuke Chinda conducted a study of 473 healthy women and found that H. pylori is not a significant risk factor for osteopenia [28]. In our study, we analysed the relationship between H. pylori infection and osteoporosis. We found a significant relationship between H. pylori infection status and bone density in premenopausal females but not in males. We suspect this may be due to the difference in the aetiology of osteoporosis between males and females. However, we did not find any other studies on this, and it requires more systematic research for analysis.

After analysing the differences between males and females, we found that there were significant differences in BMI, WHR, and TG in the study population. This study provides evidence for follow-up research on sex differences in the relationship between H. pylori and osteoporosis.

Most studies hold the view that obesity is related to osteoporosis [29]. It is generally believed that obesity may be a protective factor against bone loss and osteoporosis [30]. However, the effect of obesity remains unclear. On the one hand, obesity has traditionally been considered positive for bone because of the beneficial effect of mechanical loading [31]. On the other hand, people hold the view that BMI may harm BMD. Osteoblasts and adipocytes both stem from marrow mesenchymal stromal cells. Osteoblasts and adipocytes are in a competitive relationship, and an increase in adipocytes will inhibit osteoblasts [30]. In our study, there was a significant relationship between BMI and osteoporosis. Increased BMD levels in obese people may be associated with increased mechanical loading and strain; this is a complicated problem that cannot be generalized.

In our study, we found that H. pylori infection is associated with a decrease in bone density. The possible reasons are as follows: First, H. pylori infection may cause systemic inflammation and increase the production of tumour necrosis factor-α, interleukin-1, and interleukin-6 [11]. These cytokines are directly involved in the reduction of BMD. We found that HCY is related to osteoporosis, which supports this hypothesis. Second, osteoporosis may be related to a decrease in vitamin B12 levels [32]. Serin et al.’s study examined 145 patients without atrophy, erosions or ulcers, and they found that the histopathological scores for both antral and corpus H. pylori density and inflammation were significantly inversely associated with serum vitamin B12 levels [13]. In our study, although we did not find a significant relationship between B12 and osteoporosis, we still support the relevant theory. The absence of our results may be due to a lack of sufficient data and the influence of confounding factors. Last but not least, most patients chronically infected with H. pylori manifest pangastritis with reduced acid secretion due to bacterial virulence factors, inflammatory cytokines, and various degrees of gastric atrophy [33]. Calcium is ionized in acidic conditions and absorbed in the small bowel. Therefore, in either hypochlorhydria or achlorhydric stomachs, calcium absorption is impaired [12]. Moreover, the long-term use of acid suppressants, for example, proton pump inhibitors, may lead to osteoporosis or a decrease in BMD. Limited experimental evidence indicates that PPI may influence calcium absorption, leading to compensatory physiologic responses, including secondary hyperparathyroidism, which may cause an increase in the rate of osteoclastic bone resorption [34]. The results showed that calcium had a trend, though it was not statistically significant (*P* = 0.076), with osteoporosis. Our results do not support the theory that there is a correlation between Ca and osteoporosis, but it may be that Helicobacter pylori infection may cause calcium absorption damage and affect BMD. We did not analyse vitamin D levels, which could affect both bone homeostasis and the inflammatory state [35]. Although H. pylori infection causing a decrease in bone density is supported by most researchers, the effect of early eradication therapy is still insufficient. Replogle ML holds the view that early eradication therapy may eliminate chronic inflammation from H. pylori [36]. Some articles have also reported an improvement in B12 levels after complete eradication [13, 37], which requires further investigation.

Despite its relevant findings, our study had several limitations. First, because most patients cannot remember the time of HP infection accurately, we were not able to obtain the time of HP infection, so different infection times may have had an impact on the results. Second, we did not collect vitamin D data, the sample size of our data was not large enough, and the study population only included participants from Beijing Shijitan Hospital, meaning that there might have confounding factors because of differences in the distribution of hospital study populations. Further large-scale studies in the general population are needed to validate our results. Third, the study participants were all Chinese, and the findings might not be generalizable to other ethnic populations. In addition, we only found some differences between men and women but failed to further explore them.

## Conclusions

H. pylori is associated with osteoporosis in premenopausal females. BMI and HCY are related to osteoporosis in premenopausal females. Chronic inflammation may be involved in the relationship between H. pylori and osteoporosis.

## Supplementary information

**Additional file 1.**

## Data Availability

The datasets analyzed during the current study are not publicly available because it includes the study population personal information which is illegal to open but are available from the corresponding author on reasonable request.

## Abbreviations

DXA: Lumbar dual-energy x-ray absorptiometry
ORs: Odds ratios
CIs: Confidence intervals
H. pylori: Helicobacter pylori
SERMs: Selective estrogen receptor modulators
proGRP: Pro-gastrin-releasing peptide
BMI: Body mass index
WHR: Waist-to-hip ratio
BMD: Bone mineral density
WHO: World health organization
SD: Standard deviation
TC: Total cholesterol
TG: Triglycerides
UA: Uric acid
LDL-C: Low-density lipoprotein cholesterol
Ghb: Glycosylated haemoglobin
HDL-C: High-density lipoprotein cholesterol
GLU: Glucose
PG: Pepsinogen
CA: Calcium
DP: Diastolic blood pressure
Hb: Haemoglobin
SP: Systolic blood pressure
C13: ^13^C breathing test positive
