# Supplementary material for: H. Pylori is related to osteoporosis but only in premenopausal female: a cross-sectional study
Source: BMC Musculoskelet Disord. 2020 Aug 18;21:559. doi: 10.1186/s12891-020-03586-7 (PMC7433125; doi:10.1186/s12891-020-03586-7)
Supplement: Supplementary file 1 — Additional file 1. [file 12891_2020_3586_MOESM1_ESM.docx]

**Table1** the relationship between the H. pylori infection and the BMD in different gender

| Age cut point |  | H. pylori infection (-) | H.pylori infection (+) | *P-value* |
| --- | --- | --- | --- | --- |
| 50-52 | Female | 16 | 16 | 0.063 |
|  | Male | 32 | 17 | 0.157 |
| 52-54 | Female | 21 | 5 | **0.011** |
|  | Male | 39 | 21 | 0.877 |
| >54 | Female | 3 | 3 | - |
|  | Male | 5 | 7 | 0.682 |

*Bold indicates statistically significant values.*
